# Suicidality risk of cancer caregivers: a systematic review and meta-analysis of observational studies

**DOI:** 10.1080/07853890.2026.2624214

**Published:** 2026-02-02

**Authors:** Xia-Yin Zhu, Lu-Yi Hu, Min-Ya Jin, Tao-Hsin Tung

**Affiliations:** a Department of Hematology, Taizhou Hospital of Zhejiang Province Affiliated to Wenzhou Medical University, Linhai, Zhejiang, China; b Department of Hematology, Enze Hospital Affiliated to Hangzhou Medical College, Linhai, Zhejiang, China; c Department of Clinical Laboratory, Taizhou Hospital of Zhejiang Province Affiliated to Wenzhou Medical University, Linhai, Zhejiang, China; d Evidence-Based Medicine Center, Taizhou Hospital of Zhejiang Province Affiliated to Wenzhou Medical University, Linhai, Zhejiang, China

**Keywords:** Cancer, caregivers, suicidality, observational study, meta-analysis

## Abstract

**Background:**

The increase in the number of global cancer cases is expected to lead to a greater caregiving burden. However, few studies have investigated the risk of suicidality among cancer caregivers.

**Objective:**

The aim of this systematic review was to explore the risk of suicidality among cancer caregivers.

**Methods:**

For this systematic review, a comprehensive search of the PubMed, Embase, Cochrane, Web of Science databases, PsycINFO and Scopus databases was conducted from inception to 16 July 2025. A meta-analysis was performed to determine the odds ratios (ORs) for case-control and cross-sectional studies and the adjusted hazard ratios (aHRs) for cohort studies.

**Results:**

Seven studies were included (four cohort studies, one case-control study and two cross-sectional studies), and 638,188 study subjects and 4,203,957 nonexposed subjects. The meta-analyses revealed that the ORs was 1.91 (95% confidence interval [CI]: 1.46–2.49) in the case-control study, cross-sectional studies, and one cohort study which reported ORs, and the aHRs was 1.32 (95% CI: 1.16–1.50) in the three cohort studies. Caregivers of patients with highly aggressive cancers had a greater risk of suicidality (aHR = 1.66; 95% CI: 1.48–1.86). Within the first 7 years following a patient’s cancer diagnosis, the risk of suicidality among caregivers remained elevated (aHR = 1.42; 95% CI: 1.04–1.94).

**Conclusion:**

The results indicate the need for both clinical and societal awareness to reduce the risk of suicidality among cancer caregivers, especially those with highly aggressive cancers.

**Trial registration:**

Registered prospectively in PROSPERO (https://www.crd.york.ac.uk/prospero/) with the registration number (PROSPERO CRD420251011926) and date of registration(15/03/2025).

## Introduction

Cancer diagnosis and treatment not only alter a patient’s behaviour and emotional and physical conditions but also impact the patient’s entire family [1]. The primary care setting for cancer patients has transitioned from hospitals to homes because of shorter hospital stays, increased availability of outpatient treatments, longer survival rates, and patients’ preferences for home care [2]. Currently, approximately six million individuals provide care to people diagnosed with cancer [3]. With population ageing, the global cancer burden is projected to continue rising, with an estimated 28 million new cancer cases in 2040 [4]. As a result, more caregivers, particularly cancer caregivers, will need to take care of patients’ everyday needs [5].

Close connections often cause existential distress for both patients and caregivers [6]. Cancer caregivers, sometimes called ‘hidden patients’, frequently experience overlooked distress [7]. A cancer diagnosis causes significant psychological distress not only for patients but also for their loved ones [8,9]. A previous meta-analysis revealed anticipatory grief among caregivers of people with chronic illness, with a pooled prevalence rate of 24.78% (95% CI 19.04% to 30.99%) [10]. Another cross-sectional study revealed that cancer caregivers face significant burdens, especially in terms of emotional, physical, and financial burdens, with 75.6% experiencing mental health issues such as anxiety and depression, 43.5% dealing with high financial costs, and 33% suffering negative physical impacts [11]. Research has shown that among cancer caregivers, older age, male sex, poor health status of the family members during caregiving, preexisting mental illness, insufficient social support and intense caregiving burden are significantly related to suicidal ideation [12]. Compared with the general population, spouses of the deceased and other bereaved cancer caregivers, especially those with insufficient preparedness for bereavement, show higher rates of suicidal ideation [13]. However, an alternative perspective suggests there is no altered risk of parental death by suicide at any time following a child’s cancer diagnosis [14]. Moreover, the risk of suicidality among family caregivers of patients with different types of cancer varies [15]. In 2024, Chen et al. conducted a meta-analysis and revealed a significantly high prevalence of suicidal ideation, suicide, and self-harm behaviour among cancer caregivers, especially those at higher risk, including caregivers older than 50 years and spouses [16]. However, research examining the risk of suicidality among cancer caregivers remains notably limited.

‘Suicidality’ is used as an umbrella term that encompasses suicide ideation, suicide attempt, and completed suicide, which are understood to lie on a continuum; as a result, large population-based studies often combine these endpoints into a single composite outcome to maximise statistical power and clinical relevance [17–19]. Although previous studies have examined the psychological stress experienced by cancer caregivers, few have quantified suicidality risk in this population [20–22]. Our aims are to conduct a systematic review to evaluate the risk of suicidality among cancer caregivers and to create evidence-based strategies to identify high-risk individuals for appropriate interventions.

## Methods

### Literature search

We searched the PubMed, Embase, Cochrane Library, Web of Science, APA PsycINFO and Scopus databases from inception to 16 July 2025 with no language restrictions applied, and used backwards and forwards searches to find more articles. The search strategy was as follows: (Tumor* OR Cancer* OR Malignanc* OR Neoplas* OR Malignant Neoplasm* OR Benign Neoplasm*) AND (Suicid* OR Suicidality OR Self-harm OR Self-mutilation OR Self-Injurious* OR Suicide attempt* OR Suicide death* OR Suicide behavior* OR Suicidal ideation* OR Suicide prevention) AND (Carer* OR Family Caregiver* OR Spouse Caregiver* OR Informal Caregiver* OR Mother* OR Father* OR Grand* OR Sibling* OR Brother* OR Sister* OR Bereave* OR Bereavement OR Famil* OR Family Member* OR Filiation OR Kinship Network* OR Relative* OR Offspring OR Parent* OR Step-Parents* OR Healthcare Work* OR Married Person* OR Spous* OR Husband* OR Wives OR Wife OR Domestic Partner* OR Nuclear Famil* OR Daughter* OR Son). The search strategy is detailed in Table S1.

We conducted this systematic review and meta-analysis in accordance with the 2020 Preferred Reporting Items for Systematic Reviews and Meta-Analyses (PRISMA) statement and have provided the completed PRISMA checklist as a supplementary file (Supplementary File). The protocol for this systematic review and meta-analysis was registered on PROSPERO (registration number: CRD420251011926).

### Study selection

We included studies that met the following inclusion criteria (1): cohort, cross-sectional, or case-control design (2); the exposed group comprised cancer caregivers and the nonexposed group population-based controls; and (3) reported data on composite outcomes of suicide deaths, attempts or ideation [17–19]. Titles and abstracts were screened; studies with unrelated topics or those that were reviews, meta-analyses, or conference abstracts (typically lacking sufficient detail) were excluded. Full texts were retrieved for potentially eligible studies. ZXY and HLY independently screened and selected studies; disagreements were referred TTX for adjudication. All disputes over study inclusion or data interpretation were then resolved through consensus among the entire team.

### Data extraction and quality assessment

From the included studies, we gathered the following information: the first author, year of publication, country, number of people, category of caregiver, age at cohort entry, sex, timing after diagnosis, cancer aggressiveness, and family history of psychiatric disorders. Specifically, cancer aggressiveness was defined as follows:

High aggressiveness was assigned to cancers with low 5-year survival rates, including lung, esophagus, liver, pancreas, ovary, and unknown/ill-defined cancers [14,15]. For prostate cancer, high aggressiveness was further defined by clinical stage (T3/T4), Gleason score (≥8), and PSA level (≥20 ng/mL) at diagnosis. Distant metastases (M1) or very high PSA levels (≥100 ng/mL) indicated advanced disease [23]. The primary outcomes of interest included the occurrence of suicide attempts, suicide deaths, or suicide ideation. ZXY and HLY separately collected data from each report and evaluated the bias risk of the included studies with the Newcastle-Ottawa Scale (NOS), and three aspects were assessed: selection of study groups, comparability, and exposure, differences were resolved *via* discussion with TTX. For cohort and case-control studies, we used the original NOS scales. (https://www.ohri.ca/programs/clinical_epidemiology/oxford.asp). For cohort studies (Total score: 9 points) and case-control studies(Total score: 8 points), the scale categorizes quality into three levels based on scores: low quality (0–3 points), moderate quality (4–6 points), and high quality (≥7 points), with 9 points represented the highest methodological quality [24]. For cross-sectional studies, we used a standardized tool adapted from the Newcastle-Ottawa Scale (NOS) which developed by Modesti et al. which has been widely adopted in systematic reviews across various fields, and which divides quality into four grades: very good (9–10 points), good (7–8 points), satisfactory (5–6 points), and unsatisfactory (0–4 points) [25,26]. See Appendix 1 for more details.

### Analysis

For the meta-analysis, we utilized Review Manager 5.3 software from The Nordic Cochrane Centre, The Cochrane Collaboration, 2014. We report the risk of outcomes as the adjusted hazard ratios (aHRs) for three cohort studies, and the odds ratios (ORs) for cohort, case-control, and cross-sectional studies, all with 95% CIs. However, one included study, despite being a cohort design, reported only odds ratios (ORs) and did not provide hazard ratios (aHRs) or sufficient data for their calculation. Consequently, this study was pooled with case-control and cross-sectional studies in the OR-based meta-analysis. Heterogeneity was assessed *via* the I^2^ statistic, which measures the variability between studies attributable to heterogeneity rather than chance, with an I^2^ value ≥50%, indicating significant heterogeneity [27]. If I^2^ was 50% or higher, we used a random effects model. If I^2^ was less than 50%, we used a fixed-effects model. Where I^2^ was ≥ 50%, we conducted subgroup analyses by cancer aggressiveness, caregiver sex, age, and time since diagnosis to explore heterogeneity. Funnel plots were utilized to assess potential publication bias. We also performed a sensitivity analysis by excluding one study at a time and recalculating the pooled effect size to assess the robustness of the main findings.

## Results

### Characteristics of included studies

**Figure 1.**
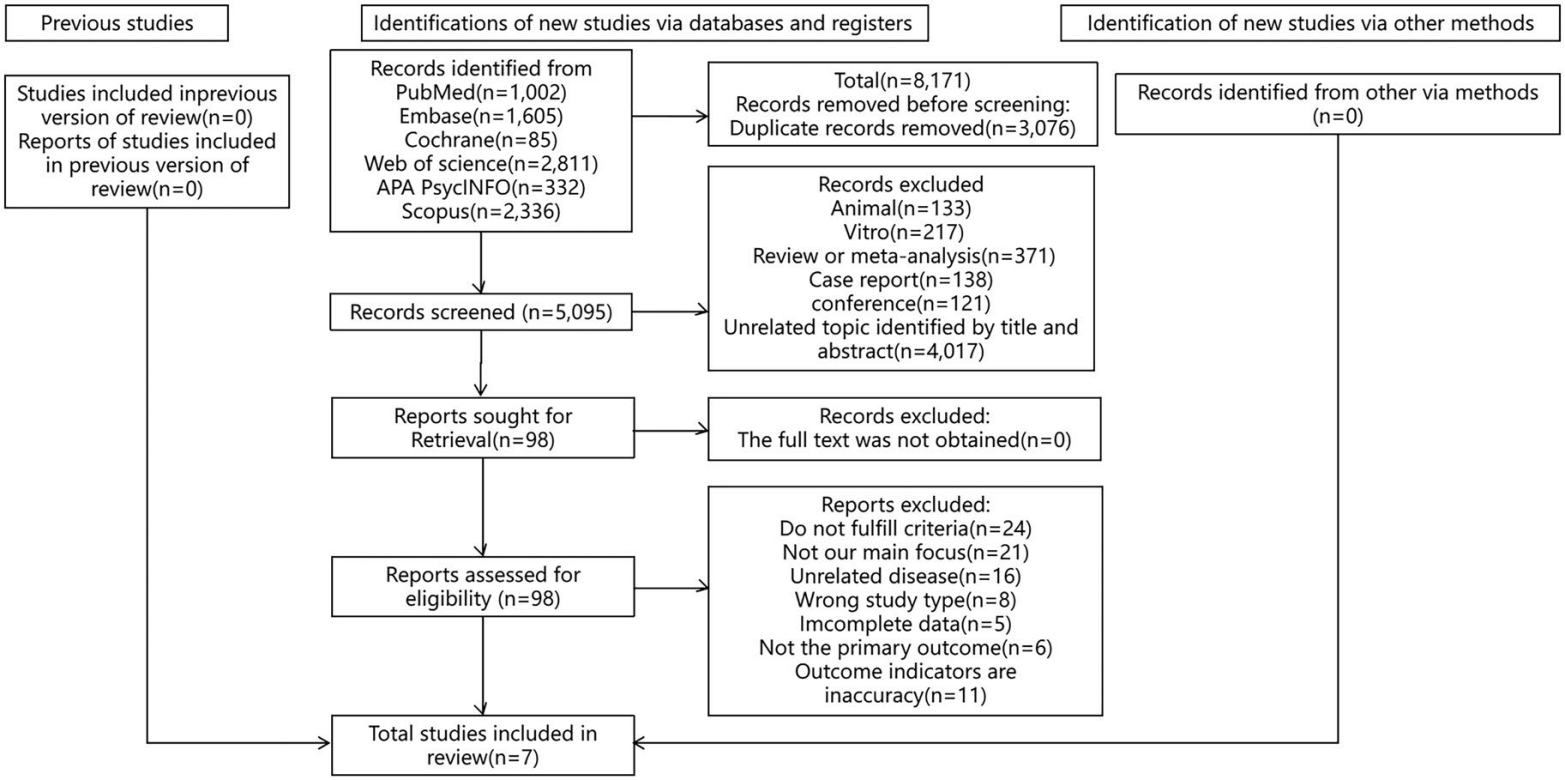
PRISMA flow diagram for details on the process of selecting studies for the present meta-analysis and systematic review. N: Number of included studies.

As illustrated in Figure 1, 8,171 records were identified in the search. After deduplication, 5,095 records remained, of which 5,088 were excluded because they failed to meet the selection criteria. Seven observational studies—four cohort, one case-control, and two cross-sectional studies—were ultimately included. All seven studies were included the systematic review and provided data for the meta-analyses. These studies included 638,188 exposed individuals (636,947 family caregivers of cancer patients from the four cohort studies and 1,241 family caregivers of cancer patients from the one case-control and two cross-sectional studies) and 4,203,957 nonexposed individuals (4,200,111 individuals from the cohort studies and 3,846 individuals from the case-control and cross-sectional studies).

Table 1 and Table 2 summarised the characteristics of the included studies; Table 3 showed the results of the Newcastle–Ottawa quality assessments, all studies were rated as high quality (≥7 points). The results of the sensitivity analyses were shown in Figure S1, and the results of the funnel plot evaluations were shown in Figure S2.

**Table 1.** Characteristics of included studies.

Authors, year, country, Number	Category of the caregivers	Suicide attempts, Suicide deaths, Suicide ideation	Age at cohort entry years	Gender	Timing after diagnosis	Cancer aggressiveness	Family history of psychiatric disorder
Qianwei Liu et al. [14], 2024, Denmark or Sweden, exposed: 106,005; matched unexposed: 1,060,050	Parents	aHR(95%CI) suicide attempts: ≤7 years: 1.15(1.03,1.28) >7 years: 0.86(0.75,0.98)	aHR(95%CI) suicide attempts: ≤7 years: <40:1.27(1.06,1.52) 40-60:1.18(1.02,1.36) >60:0.79(0.56,1.09) >7 years: <40:0.96(0.81,1.12) 40-60:0.73(0.57,0.93) >60:0.53(0.19,1.45)	aHR(95%CI) suicide attempts: ≤7 years male: 1.04(0.88,1.24) female: 1.23(1.07,1.41) >7 years male: 0.77(0.62,0.95) female: 0.93(0.79,1.10)	aHR(95%CI) suicide attempts: ≤7 years: 1.15(1.03,1.28) >7 years: 0.86(0.75,0.98)	aHR(95%CI) suicide attempts: ≤7 years: Low aggressiveness: 1.10(0.89,1.37) Medium aggressiveness: 1.13(1.10,1.28) High aggressiveness: 1.60(1.05, 2.43)	aHR(95%CI) suicide attempts: ≤7 years Yes: 0.98(0.76,1.25) No: 1.19(1.06,1.34) >7 years Yes: 0.98(0.72,0.97) No : 0.84(0.72,0.97)
Qianwei Liu et al. [15], 2024, Denmark, exposed: 409338; Unexposed: 2046682	Spouse	aHR(95%CI) suicide attempts: 1.28(1.23–1.34) spouse death: 1.57(1.49–1.66)	aHR(95%CI) suicide attempts: 18-39: 1.10(0.94,1.29) 40-59: 1.34(1.25,1.44) 60-79: 1.28(1.20,1.35) ≥80: 1.11(0.89,1.39) suicide deaths: 18-39: 1.79(1.19,2.70) 40-59: 1.40(1.22,1.61) 60-79: 1.49(1.33,1.68) ≥80: 1.50(1.05,2.15)	aHR(95%CI) suicide attempts: male: 1.42(1.33–1.52) female: 1.21(1.14,1.27) suicide deaths: male: 1.39(1.26–1.54) female: 1.68(1.45,1.96)	aHR(95%CI) <1 years: suicide attempts: 1.45 (1.27–1.66) suicide deaths: 2.56 (2.03–3.22)	aHR(95%CI) suicide attempts: spread or an advanced stage: 1.66(1.46–1.89); Localized stage: 1.10(0.97–1.25) suicide deaths: advanced stage 1.61(1.21–2.15)	aHR(95%CI) suicide attempts: Yes: 8.61(7.97–9.30) No: 1.25(1.19–1.32)
Casey Crump et al. [23], 2023, Sweden, exposed: 121530 Unexposed: 1093304	Spouse	aHR(95%CI) suicide deaths: high-risk PC: 1.39(0.91–2.11) low- or intermediate-risk PC: 1.32(0.88–1.99)	aHR(95%CI) suicide deaths among high-risk PC: <60 years: 7.55(2.20–25.89) 60-69 years: 2.23(0.80–6.21) 70-79 years: 0.90(0.38–2.11) ≥80 years: 0.35(0.04–3.11) low- or intermediate-risk PC: <60 years: 0.69(0.25–1.95) 60-69 years: 2.14(0.98–4.70) 70-79 years: 2.05(0.69–6.09) ≥80 years: NE	aHR(95%CI) suicide deaths: female high-risk PC: 1.39(0.91–2.11) low- or intermediate-risk PC: 1.32(0.88–1.99)	aHR(95%CI) suicide deaths high-risk PC: <2 years: 0.64(0.18–2.23) 2-5 years: 2.27(1.08–4.75) 5-10 years: 1.11(0.52–2.36) ≥10 years: 1.33(0.48–3.64) low- or intermediate-risk PC: <2 years: 0.89(0.29–2.71) 2-5 years: 0.64(0.26–1.59) 5-10 years: 1.33(0.65–2.74) ≥10 years: 4.21(1.63–10.83)	aHR(95%CI) suicide deaths: distant metastases: 2.38(1.08–5.22) locally advanced 0.89(0.48–1.64)	NA
Kyae Hyung Kim et al. [35], 2022, Korea, exposed: 266(13); Unexposed: 3163(130)	Adolescent	OR(95%CI) suicide attempts: 1.20(0.65 − 2.23)	NA	NA	aOR(95%CI) suicide attempts:≤1 years: 2.96(1.00 − 8.83) 2-5 years: 1.04(0.37–2.91) ≥5 years: 0.95(0.34–2.66)	NA	NA
Tove Bylund Grenklo et al. [50], 2013, Sweden, exposed: 622(38); Unexposed: 330(13);	Adolescent	OR(95%CI) suicide attempts: 1.6 (0.8–3.0).	NA	aOR(95%CI) suicide attempts: male 1.5(0.4–5.7) female 1.6(0.8–3.4)	NA	NA	NA
Vanessa Jantzer et al. [1], 2013, Germany, Exposed: 74(8); Unexposed: 75(2)	Adolescent	OR(95%CI) suicide attempts: 4.01(0.62–26.08)	NA	NA	NA	NA	NA
Jong Im Song et al. [13], 2012, Korea, Exposed: 353(109); Unexposed: 353(58)	Bereaved family members	OR(95%CI) suicide idea: 2.27(1.58 - 3.25)	NA	NA	NA	NA	NA

aHR: Adjusted hazard ratio; OR: odds ratio; CI: confidence interval; PC: Prostate Cancer; NA: not applicable; NE: Not Evaluated.

**Table 2.** Study characteristics and suicidality assessment.

Study (year)	Article type	Cancer patients’ age group	Caregiver relationship	Timing of suicidality assessment	Suicidality definition/measurement
Qianwei Liu et al. 2024 [14]	Cohort study	Child (≤18 y) and Adult (≥18 y)	Parent	Lifetime + bereavement	ICD-7 and ICD-10 in the Danish Cancer and ICD-7 in Swedish Cancer (register)
Qianwei Liu et al. 2024 [15]	Cohort study	Adult (≥18 y)	Spouse	Lifetime + bereavement	ICD-7 and 10th Danish revisions (register)
Casey Crump et al. 2023 [23]	Cohort study	Adult (prostate cancer)	Spouse	During parental illness	ICD-10 × 60-X84, Y10-Y34(register)
Kyae Hyung Kim et al. 2022 [35]	Cross-sectional study	Adult(≥18 y)	Adolescent child	During parental illness	Single yes/no question on suicidal thoughts
Tove Bylund Grenklo et al. 2013 [50]	Case-control study	Adult(≥18 y)	Bereaved parent	Bereavement	Self-injury & suicide attempt by questionnaire
Vanessa Jantzer et al. 2013 [1]	Cohort study	Adult(≥18 y)	Adolescent child	During parental illness	Paykel Suicide Scale (single item)
Jong Im Song et al. 2012 [13]	Cross-sectional study	Adult(≥18 y)	Bereaved spouse	Bereavement (2–6 mo post-loss)	Questionnaire of yes/no on suicidal thoughts item according to KNHANES

**Table 3.** Quality assessment of included studies using the Newcastle-Ottawa scale (NOS).

Source	Selection	Comparability	Exposure	Total NOS score	Overall quality	Notes
**Cohort studies**						
Qianwei Liu et al. [14], parents, 2024	4	2	3	9	high	—
Qianwei Liu et al. [15], Spouse, 2024	4	2	3	9	high	—
Casey Crump et al. [23], 2023	4	2	3	9	high	—
Vanessa Jantzer et al. [1], 2013	4	2	2	8	high	Exposure (3) Non-response rate
**Case-control study**						
Tove Bylund Grenklo et al. [50], 2013	4	2	3	7	high	Exposure (2) Non-Response Rate
**Cross-sectional studies**						
Kyae Hyung Kim et al. [35], 2022	4	2	2	8	good	Selection (3) Non-respondents Exposure (2) Self-reported outcomes
Jong Im Song et al. [13], 2012	5	2	3	10	very good	—

For cohort and case-control studies: High quality: NOS score (≥7); Moderate quality: NOS score (4-6) and Low quality: NOS score (≤3) [24].

For cross-sectional studies: very good quality (≥9), good quality (7-8), satisfactory quality (5-6), and unsatisfactory studies (≤4 points) [25,26].

### Main pooled estimates

**Figure 2.**
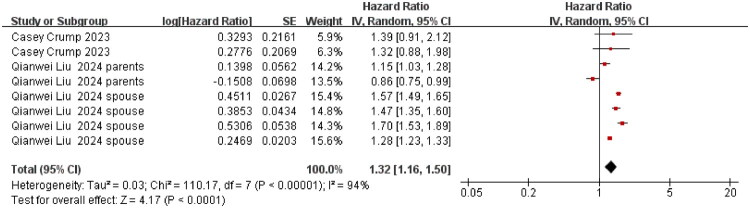
Suicidality risk among cancer caregivers: aHRs in cohort studies. The summary effect size is represented by the diamond, CI: confidence interval; SE: standard error, Heterogeneity was assessed using the I^2^ statistic.

**Figure 3.**
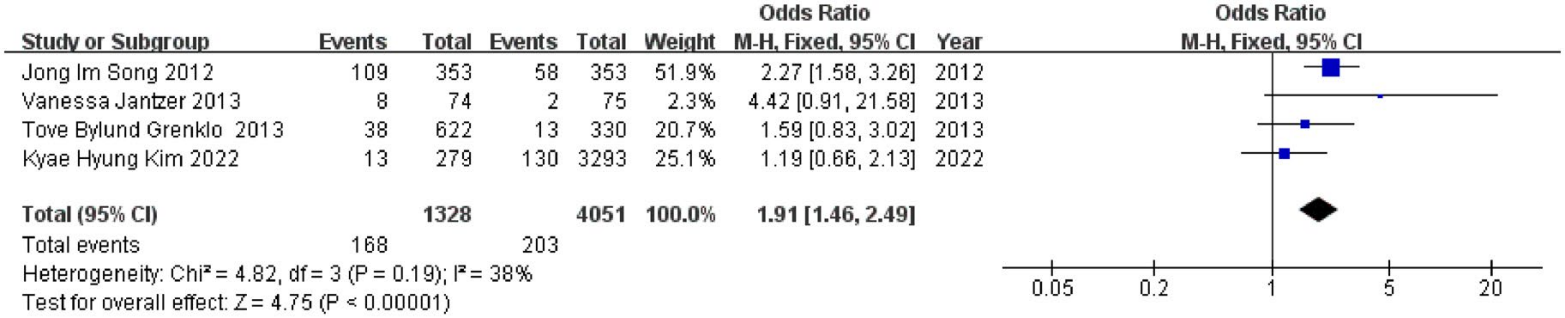
Suicidality risk among cancer caregivers: ORs in mixed studies. The summary effect size is represented by the diamond, CI: confidence interval; SE: standard error, Heterogeneity was assessed using the I^2^ statistic.

**Figure 4.**
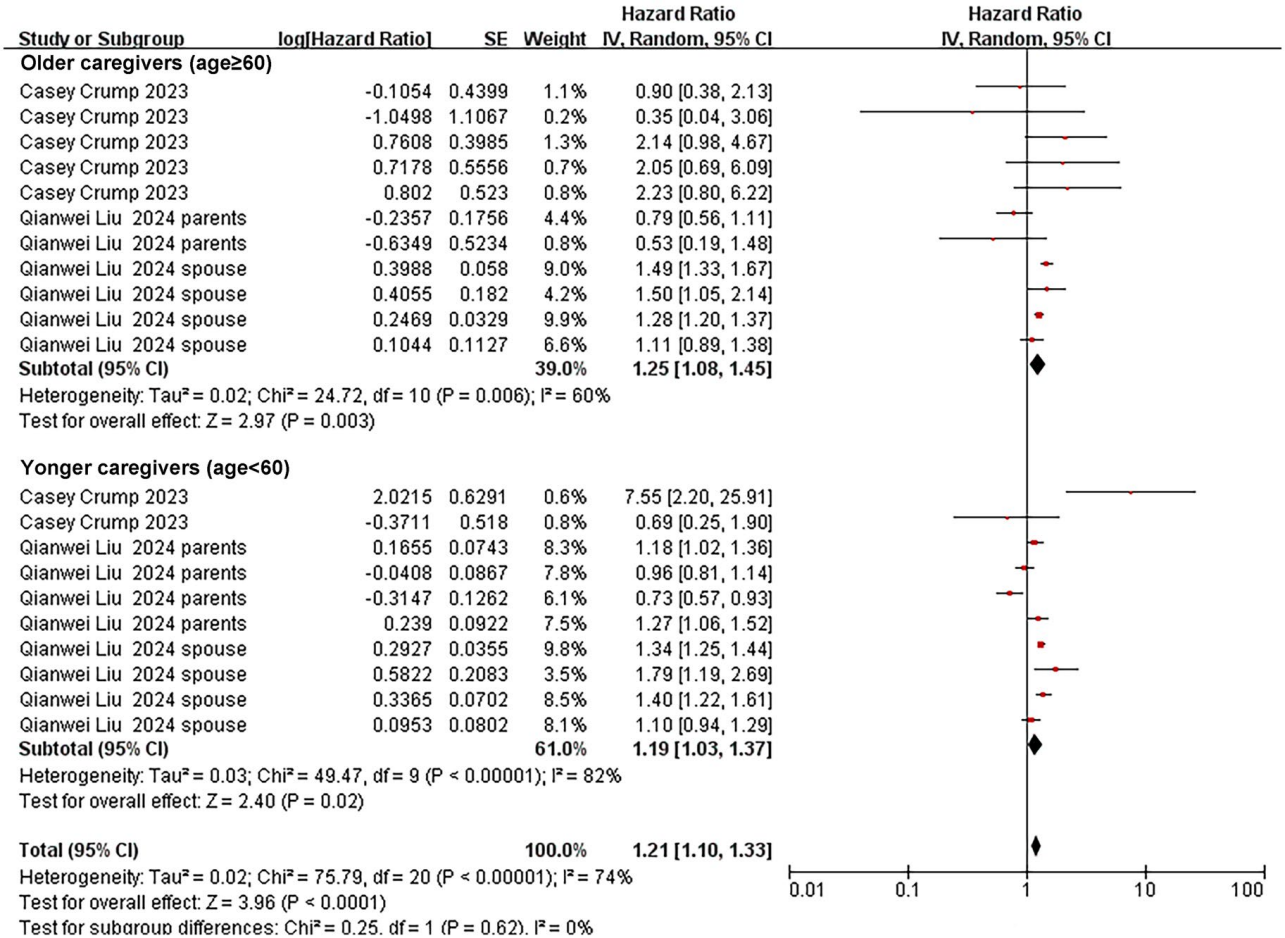
Association between age (≥60 vs. <60 years) and suicidality risk among cancer caregivers. The summary effect size is represented by the diamond, CI: confidence interval; SE: standard error, Heterogeneity was assessed using the I^2^ statistic.

**Figure 5.**
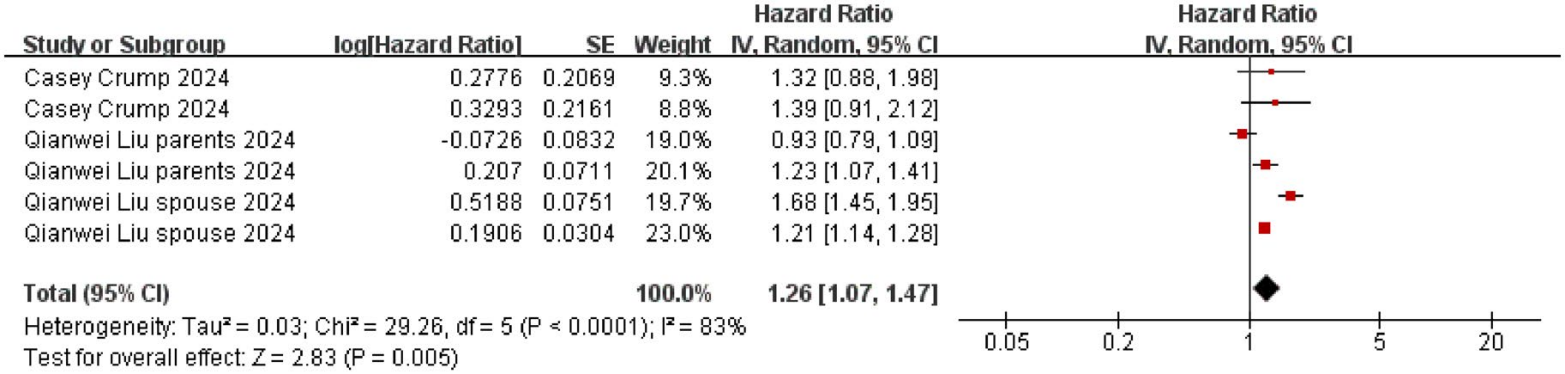
Association between female sex and suicidality risk among cancer caregivers. The summary effect size is represented by the diamond, CI: confidence interval; SE: standard error, Heterogeneity was assessed using the I^2^ statistic.

**Figure 6.**
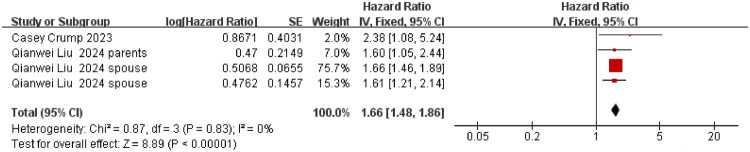
Association between highly aggressive cancer and suicidality risk among cancer caregivers. The summary effect size is represented by the diamond, CI: confidence interval; SE: standard error, Heterogeneity was assessed using the I^2^ statistic.

**Figure 7.**
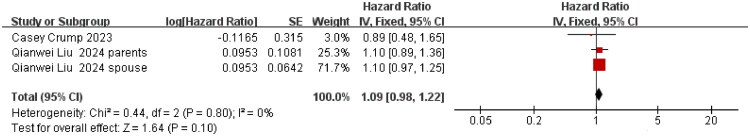
Association between low aggressive cancer and suicidality risk among cancer caregivers. The summary effect size is represented by the diamond, CI: confidence interval; SE: standard error, Heterogeneity was assessed using the I^2^ statistic.

**Figure 8.**
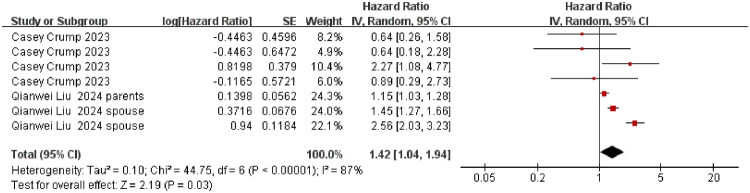
Association between diagnosis within 7 years and suicidality risk among cancer caregivers. The summary effect size is represented by the diamond, CI: confidence interval; SE: standard error, Heterogeneity was assessed using the I^2^ statistic.

Our meta-analysis revealed that family caregivers of cancer patients exhibited a significantly elevated risk of suicidality (including suicide deaths and attempts), with an aHR of 1.32 (95% CI: 1.16–1.50; *p* < 0.001), as shown in Figure 2. Substantial between-study heterogeneity was evident (I^2^ = 94%). Subgroup analyses (Figures 2–8) and the funnel plot in Figure S2 confirmed the robustness of the overall estimate.

With respect to case-control studies, two cross-sectional studies, and one cohort study that only reported OR, as shown in Figure 3, low heterogeneity (I^2^ = 38%) supported the use of a fixed-effects model, and the pooled analysis revealed a clear and significant increase in suicidality among caregivers, with the odds nearly doubled in these individuals (OR = 1.91; 95% CI 1.46–2.49; *p* < 0.001). The sensitivity analyses, as shown in Figure S1, and the funnel plots, as shown in Figure S2, confirmed the stability of these results.

### Heterogeneity and publication bias

Three cohort studies, that reported aHR as outcome, exhibited pronounced heterogeneity (I^2^ = 94%). We confirmed the robustness of the overall estimate by applying a random-effects model and conducting subgroup analyses stratified by cancer aggressiveness, caregiver sex, age, and time since diagnosis. Moreover, funnel plots for each comparison were displayed in Figure S2, and visual inspection revealed a largely symmetrical distribution of effect estimates around the pooled effect, with no overt clustering of small studies towards either side. This qualitative assessment provided reassurance against meaningful small-study effects or publication bias; however, the small number of studies limited the statistical power to formally assess publication bias.

Case-control and cross-sectional studies, as well as one cohort study that only reported OR, analyses demonstrated low heterogeneity (I^2^ = 38%). When sensitivity analyses were restricted to meta-analyses comprising more than three studies, leave-one-out iterations revealed that the pooled effect remained statistically significant in every scenario (aHR 1.51–2.15), underscoring the robustness of the primary findings (Figure S1).

### Subgroup analyses

We performed subgroup analyses of the three cohort studies that reported aHR, stratified by age, sex, cancer aggressiveness, and time elapsed since diagnosis to examine whether the link between caregiving and suicidality varied across patient and caregiver characteristics.

The pooled aHR for suicidality (suicide attempts and deaths) was 1.19 (95% CI 1.03–1.37; *p* = 0.02; I^2^ = 82%) for caregivers younger than 60 years; for those aged ≥ 60 years, the aHR was 1.25 (95% CI 1.08–1.45; *p* = 0.003; I^2^ = 60%). We further conducted a test for subgroup differences between caregivers <60 years and ≥60 years. The result showed no significant difference in suicidality between the two age groups (*p* = 0.62) (Figures 4). Restricting the analysis to female caregivers yielded an aHR of 1.26 (95% CI 1.07–1.47; *p* = 0.005; I^2^ = 83%) (Figure 5). Caring for patients with highly aggressive malignancies was associated with an aHR of 1.66 (95% CI 1.48–1.86; *p* < 0.001; I^2^ = 0%), whereas the estimate for caring for patients with low-aggressive malignancies was 1.09 (95% CI 0.98–1.22; *p* = 0.10; I^2^ = 0%) (Figures 6 and 7). The aHR was 1.42 (95% CI 1.04–1.94; *p* = 0.03; I^2^ = 87%) for caregivers assessed within seven years of their patient’s cancer diagnosis (Figure 8).

## Discussion

Our study revealed that cancer caregivers have a greater risk of suicidality than the general population does, highlighting the need for psychological support for family-oriented cancer patients. Our analysis further revealed an increased suicide risk in females and all-aged cancer caregivers, which remained elevated within 7 years after cancer diagnosis. These findings suggest that family caregivers of patients with invasive cancers face an increased risk of suicidality.

## Clinical implications

When a patient is diagnosed with cancer, their family caregivers are often expected to act as caregivers because of close familial bonds [28]. Our study has shown that, when considering the family as a unit, the suicide risk among cancer caregivers is greater than that in the general population. Parents of adolescents with a cancer diagnosis often experience intense emotional distress [29], and parents of children with cancer often encounter significant physical, psychological, and financial difficulties [30]. Unplanned admissions, frequent clinic visits, and decreased function make it difficult for parents of cancer patients to care for their children [31]. Research has indicated that a cancer diagnosis can lead to increased economic strain, psychological distress, and mental health issues among the caregivers [32]. Compared with the general population, mothers and siblings of cancer patients often experience a greater and more prolonged need for mental health care; therefore, providing increased psychosocial support for family caregivers both during and after cancer therapy is essential [9]. In this study, the family caregiver categories included parents, children, and spouses, highlighting the need for psychological support for all cancer caregivers. However, we did not assess the risk of suicidality in siblings of cancer patients. As siblings are a very under researched group, examining the mortality risk of all family caregiver categories, including siblings, within a family unit and identifying which specific categories have higher mortality risks may be a meaningful research direction in the future [33,34].

We conducted a meta-analysis of cancer caregivers who were females and reported that their suicide risk was greater than that of the general female population. Compared with other family caregivers, daughters are more likely to take on caregiving roles for their parents [35]. When parents are diagnosed with cancer, girls tend to experience more psychological symptoms than boys do [36]. When children are diagnosed with cancer, mothers experience heightened psychological distress and an increased risk of psychiatric disorders [37,38]. Males in the general population have fewer suicide attempts but higher completion rates than females [39,40]. Owing to limitations in data availability, we did not analyse the suicide risk of male family caregivers. Previous meta-analyses have shown that men experience greater impacts from spousal death than women do [41,42]. Research has indicated that men experience a significant increase in suicide risk following a partner’s cancer diagnosis (mortality–morbidity rate =2.90; 95% CI: 1.70–4.93) [32]. Men generally have a lower baseline risk of psychiatric disorders, and, owing to their distinct social roles and sources of emotional support, they are typically less prepared for caregiving and coping with bereavement than women are [43]. Future research could focus on analysing the suicide risk of male family caregivers and comparing it with that of female family caregivers.

Previous research has shown that suicidal ideation tends to increase with age [44]. Specifically, it is most prevalent among older adults, followed by adolescents, middle-aged adults, and young adults [44]. Other studies have revealed that cancer caregivers who are older than 50 years tend to experience higher levels of psychological distress, such as anxiety and depression, and lower quality of life, which may increase their risk of suicidal ideation [45]. Another study found that high-risk prostatic cancer(PC) was more strongly associated with depression and anxiety in partners over 80 years of age, yet was more strongly associated with suicide deaths in partners under 60 years of age [23]. In our study, we conducted separate meta-analyses for cancer caregivers older and younger than 60 years of age. We found that both age groups presented greater risks of suicidality than did the general population. Therefore, in future research, it may be more appropriate to implement protective measures for cancer caregivers across all age groups.

Our study revealed that cancer caregivers with invasive tumours had an increased risk of suicide. A Swedish study revealed that partners of men with distant metastases had a 2-fold increased risk of suicide [23]. Additionally, an elevated risk of suicide was observed among partners of men with advanced PC but not among those with low- or intermediate-risk PC [23]. After a child’s cancer diagnosis, parents face an increased risk of suicide attempts, particularly when dealing with aggressive cancer [14]. A study revealed that the spouses of patients with cancers, such as those of the oesophagus, lung, pancreas, and liver/biliary passages, which have poor prognoses, experienced the highest documented rate of suicide [46]. No associations were noted for suicide attempts or deaths in patients with nonmelanoma skin, thyroid, or other endocrine gland cancers, likely due to the relatively favourable prognosis of these cancers [15]. The level of caregiving burden can vary by the type of cancer and the stage at which it is diagnosed [47]. When cancer is diagnosed at an advanced stage, the association between a spouse’s cancer diagnosis and suicidal behaviour is stronger, which may be due to the greater disease burden or the increased fear of the spouse’s death [15]. Although our study did not distinguish which tumour subgroup had a greater survival risk among cancer caregivers, it may be meaningful to pay greater attention to those with more aggressive and advanced-stage tumours.

Parents are at increased risk of attempting suicide within the first year following a child’s cancer diagnosis [14]. Within 1 year after parental cancer diagnosis, children’s risk of experiencing suicidal thoughts, plans, or attempts increased (aOR), 2.96; 95% CI: 1.00–8.83) [35]. In Denmark, the risk of suicide attempts and deaths among spouses of cancer patients is elevated, particularly in the first year following the cancer diagnosis [15]. The risk of suicide attempts and deaths among cancer family caregivers significantly increased during the first year after diagnosis and remained elevated throughout the follow-up period [43]. In addition, research findings have indicated that the risk of suicide among men remains elevated even more than 5 years after their partner’s cancer diagnosis [41]; this highlights the significant cumulative burden faced by spousal caregivers and underscores the need for long-term support for those caring for cancer patients [41]. Our study revealed that within 7 years after cancer diagnosis, the risk of suicidality of family caregivers remained higher than that of the general population. This finding highlights the necessity for persistent community support of cancer caregivers’ health behaviours and mental health.

## Heterogeneity and publication bias in the meta-analysis

Heterogeneity represents a critical consideration in meta-analyses, as excessive levels of such variability can undermine the reliability of findings [48]. The I^2^ statistic is widely employed to quantify the percentage of total variation that stems from heterogeneity across included studies. The cohort studies that reported aHRs included in the review exhibited pronounced heterogeneity (*n* = 3, I^2^ = 94%), whereas the case-control and cross-sectional studies, as well as one cohort study that only reported OR, demonstrated low heterogeneity (*n* = 4, I^2^ = 38%). Subgroup analysis was conducted to further explore the sources of heterogeneity and revealed that between-group heterogeneity decreased after stratification by age, sex, time since diagnosis, and cancer aggressiveness. However, heterogeneity remained unavoidable.

Publication bias is defined as the failure to publish the results of a study on the basis of the direction or strength of the study findings [49]. Funnel plots were generated for each comparison and appeared broadly symmetrical around the pooled effect, qualitatively ruling out publication bias. Sensitivity analyses confirmed that the pooled effect remained statistically significant after sequential leave-one-out iterations in every instance, underscoring the robustness of the primary findings. The findings indicated that publication bias had a limited effect on the results of the meta-analysis.

In addition, Table 3 showed that all studies were rated as high quality (≥7 points) by NOS. Kyae Hyung Kim et al.(2022) scored 7 points: one point was deducted in Selection 3 as non-respondents. And another point deducted in exposure 2 as self-reported outcomes [35]. Tove Bylund Grenklo et al. (2013) scored 7 points: one point was deducted in Exposure 2, because exposure ascertainment was not blinded to outcome assessors, and comparability was limited to matching variables [50]. Vanessa Jantzer et al. (2013) received 8 points; the single point loss was due to incomplete adjustment for additional confounders beyond matching factors in Exposure 2 [1]. Three cohort studies [14,15,23] and one cross-sectional study [13] were rated as the maximum score.

## Limitations

First, it is difficult to explore the causal relationship between suicidality risk and cancer caregivers because there are only three cohort studies reported aHRs. One cohort study reported only OR; we therefore pooled it with case-control and cross-sectional studies after confirming the outcome was measured at a single time point. The quality of evidence may thus be restricted. Second, owing to the lack of information on prognostic markers of cancer in some studies, such as molecular biological markers, this approach to assessing cancer aggressiveness may not be highly accurate. Third, the instruments used to identify suicidality varied across studies. Some relied on ICD-coded mortality or hospital records [14,15,23], while others used single-item self-report questions [1,13,35,50]. No study employed more comprehensive, clinician-administered tools such as the Columbia-Suicide Severity Rating Scale (C-SSRS) [51]. The estimated bias may occur due to some grey areas, for example, mild or passive suicidal thoughts may have been missed, and ICD-coded causes of death or hospital admission can be misclassification (e.g. ‘undetermined intent’ or accidental poisoning coded as suicide). These grey areas can blur the true effect and make it harder to compare studies. Fourth, the included studies varied in both the age group of patients (adult vs. paediatric) and the timing of suicidality assessment (during caregiving vs. after patient death). Because most studies did not report these details separately, we could not estimate whether caregivers of children or bereaved caregivers face higher risk. Fifth, this study includes data from only a few countries, the results may significantly limit the generalizability, particularly given that suicidality rates and cultural attitudes toward mental health vary substantially across populations [52,53]. Further research is needed to explore how our findings apply to countries with different health care systems, cultural backgrounds, and cancer burdens. Sixth, owing to data limitations, it was not only very difficult to conduct subgroup analyses of suicidality risk among different types of caregivers, but also lack of representativeness of other family caregivers. Seventh, not all included studies distinguished between professional and family caregivers [54]. Eighth, psychiatric conditions such as major depression and schizophrenia are recognized as crucial factors influencing suicide risk [55]; however, in our study, we did not analyse whether mental illness affects suicide risk, which is one of the directions for future research. Finally, we set the 7-year window to match the longest follow-up available [14], allowing us to include as many cohorts as possible. However the timeframe is provisional and should be refined as more data emerge.

## Practical recommendations

Caregivers have diverse needs, yet these needs are rarely assessed systematically [5]. Further studies are needed to explore family-centred interventions for relatives of cancer patients [35]. Family caregivers currently lack adequate psychosocial support. The development of a comprehensive system for cancer caregivers is crucial for early detection and targeted intervention to prevent adverse outcomes such as suicide [56,57]. Systematic screening for suicide risk should be integrated into routine oncology family visits after a cancer diagnosis; this approach necessitates the collaboration of psychosocial oncology clinicians, psychologists, and psychiatrists within oncology teams [58,59].

When family caregivers express suicidal thoughts or behaviours due to anticipatory grief, immediate assessment and coordination with mental health clinicians are essential [60]. Creating a secure environment in which family caregivers can voice their emotions, address their challenges, and develop strategies to cope is essential [61]. Society should offer respite care services to provide temporary relief for caregivers by having professional caregivers step in to take over caregiving duties [62]. People are starting to realize that cancer caregivers need emotional support, but only a small fraction ever receive proven mental-health programs, integrating caregiver support into routine cancer care is still in its early days [63].

## Conclusion

Our findings highlight the need to focus on the risk of suicidality among cancer caregivers, particularly those of patients with aggressive cancers, female family caregivers, and within the first seven years after cancer diagnosis. Future research should evaluate the impact of cancer on the risk of suicidality in family units and highlight the critical need for timely interventions.

## Supplementary Material

PRISMA_2020_checklist.docx

## Data Availability

The data that support the findings of this study are available from the corresponding author, THT, upon reasonable request.

## Appendix 1. Newcastle-ottawa scale (NOS) Assessment criteria

### Cohort studies

Selection (4 points)

1. Representativeness of the exposed cohort.
2. Selection of the non-exposed cohort.
3. Ascertainment of exposure.
4. Demonstration that outcome of interest was not present at start of study.

Comparability (2 points)

1. Comparability of cohorts on the basis of the design or analysis.

Exposure (3 points)

1. Assessment of outcome.
2. Was follow-up long enough for outcomes to occur.
3. Non-response rate.

### Case-control studies

Selection (4 points)

1. Is the Case Definition Adequate?.
2. Representativeness of the Cases.
3. Selection of Controls.
4. Definition of Controls

Comparability (2 points)

1. Comparability of Cases and Controls on the Basis of the Design or Analysis.

Exposure (2 points)

1. Ascertainment of Exposure.
2. Non-Response Rate.

### Cross-sectional studies

Selection (5 points)

1. Representativeness of the sample.
2. Sample size.
3. Non-respondents.
4. Ascertainment of the exposure (risk factor) (2 points).

### Comparability (2 points)

1. The subjects in different outcome groups are comparable, based on the study design or analysis. Confounding factors are controlled.

### Exposure (3 points)

1. Assessment of the outcome.
2. Statistical test (2 points).
